# Exploring perceptions of alternative assessment and grading in graduate anatomy education

**DOI:** 10.1002/ase.2550

**Published:** 2024-12-31

**Authors:** Emily L. Dietrich, Sean C. McWatt

**Affiliations:** ^1^ Department of Anatomy and Cell Biology, Schulich School of Medicine and Dentistry Western University London Ontario Canada; ^2^ School of Kinesiology, Faculty of Health Sciences Western University London Ontario Canada; ^3^ Centre for Education Research and Innovation, Schulich School of Medicine and Dentistry Western University London Ontario Canada

**Keywords:** alternative assessment, anatomy education, feedback, formative evaluation, individualized assessment, student perceptions, thematic analysis

## Abstract

Alternative assessment approaches, such as pass/fail and feedback‐based designs, aim to reduce academic stress and foster deeper learning. Few studies have examined feedback‐based evaluation in formative settings in medical education, but none among graduate anatomy students. This exploratory study investigated the impact of feedback‐based versus quiz‐based assessments on graduate students' academic stress, motivation, and perceived learning quality in an anatomy course. Nine students were interviewed to discern perceptions of the impact of the different assessment types. Four instructors were interviewed to establish the philosophies behind their chosen assessment style, and their responses were compared to the perceptions of their students. Inductive thematic analyses of student interviews yielded multiple themes regarding considerations for the influence of assessment on academic‐related anxiety and motivation: (1) communicating clear goals and expectations, (2) instructor approachability and individualized assessment, and (3) alignment between perceived effort and outcomes. Faculty interviewees demonstrated intentions to (1) promote non‐technical skills, (2) provide authentic experiences, and (3) emphasize individualized assessment. However, there was some misalignment between instructors' goals and students' experiences, exacerbating students' stress and reducing their motivation. Feedback‐based assessments were preferred for their perceived individualized nature and facilitation of deeper learning. Findings indicated that implementing feedback‐based approaches, while ensuring constructive alignment, could reduce stress for graduate anatomy students. Furthermore, clear communication and instructor approachability can cultivate stronger teaching presence, which may enhance motivation, engagement, and the quality of learning outcomes. Implementing formative feedback‐based assessments may, therefore, be an effective strategy to reduce stress and improve learning experiences for graduate‐level anatomy students.

## INTRODUCTION

Assessment and grading are foundational pillars of education systems worldwide, beginning from early elementary years and extending through to graduate and professional education. Historically, graded assessments have served primarily as tools for internal communication between teachers and parents, as well as for students, themselves, to know where they stand academically.^1^ However, the use of grades in these contexts only provides students with information on their achievement when judged against a series of criteria, without providing tangible information regarding the potential gaps in their knowledge.^2^ With these such assessments, the majority of which tend to be more summative in nature, educators can bypass the element of feedback in favor of “feedout” in the form of grades; therefore, while students and parents receive some communication related to their academics, the grades may not be representative of the more nuanced learning process.^1^, ^3^

While grades were initially introduced as a tool for communication, they quickly became a source of extrinsic motivation toward academic success. This turned the focus of education toward feelings of competition among students, where the most “strategic” learners would focus only on the content that would merit them higher grades.^1^, ^4^ Other types of learners, particularly those with poor academic self‐efficacy, would approach assessments with the goal of “performance‐avoidance,” where they would aim to minimize negative outcomes rather than aim for positive ones; that is, they would work to avoid performing poorly, rather than learning in order to excel.^5^ Therefore, the incentive of grades was undermining the learning process, with detriments to students.^6^ Consequently, willingness to take risks, especially the risk of being wrong, was deterred by the extrinsic motivation that grades provided, inhibiting the development of lifelong learning skills.^6^

Naturally, educators have recognized a need to transition away from extrinsic motivators and redirect students' focus onto finding intrinsic motivation to learn. Gorichanaz (2024) found that students reported increased intrinsic motivation to learn in environments that had either minimal or no reliance on grades.^6^ This finding is especially relevant for students with high test anxiety (a personality trait that prompts individuals to see assessments as threatening situations) who are less motivated to learn in classrooms with strong emphasis on assessment or evaluation.^7^ Of those who experience test anxiety, over 25% will experience lower academic performance as a result.^8^ Many students have also expressed feelings that grades represent proxy metrics of their abilities or worth; hence, poorer grades can hinder their sense of self‐efficacy and can fuel their academic‐related stress.^5^, ^9^, ^10^ Therefore, to truly enhance the student experience and bring focus back to learning, some educators choose to approach grading differently.

To focus on metacognitive skill development, intrinsic motivation, and learning for the sake of learning, many instructors have begun to adopt alternative assessment practices including but not limited to self‐assessment, peer assessment, pass/fail grading, and feedback‐based assessment.^6^, ^11^, ^12^ With each of these examples, student learning, rather than performance, is the primary focus. Though these types of assessments may not always require the complete elimination of grades, they can excel in areas where traditional grading is insufficient to evaluate learning goals. For instance, by decentering grades, students have been able to engage in deeper learning, foster their intrinsic motivation, and reduce their academic‐related stress.^6^, ^12^, ^13^

Some approaches that are acutely targeted toward the learning process, rather than the product, are forms of feedback‐based assessment. This approach is centered around providing students with feedback to help them gauge their own academic performance during the learning process, which can come in different forms, depending on the needs of specific disciplines.^14^ For example, many medical programs incorporate feedback‐based formative assessment in exercises with standardized patients.^14^, ^15^ In these instances, most of the feedback provided is verbal, whereas in others, students may receive written feedback on formative assessments from their instructors.^16^ For students to perceive feedback as effective, it should relate to clearly defined criteria for performance, whether that is outlined orally or in rubrics.^17^ Incorporation of effective feedback into assessment is vital for enhancement of learning outcomes, since students commonly view assessment as the basis for what is important in their learning.^18^ Therefore, feedback‐based assessments are defined by the present authors as any assessments that prioritize and emphasize the provision of feedback to students while de‐emphasizing the numeric grade. In studies exploring assessments for which instructors have provided feedback, students have reported experiencing enhanced intrinsic motivation when the feedback they received offered insight into their “*competence*” and communicated if and where they had gaps in their knowledge.^13^ Having higher levels of intrinsic motivation is linked to academic achievement, which suggests that by reducing the prevalence of grades and instead emphasizing feedback, instructors may be able to help students achieve more.^19^, ^20^

Intrinsic motivation has additionally been found to dampen the negative effects of test anxiety on achievement; therefore, it is unsurprising that assessment approaches such as pass/fail or feedback‐based assessments have been linked to reduced student anxiety levels.^20^ These such results have been seen consistently across a variety of undergraduate‐level courses in fields including history, nursing, mathematics, organic chemistry, and exercise physiology, and benefits have also been reported at the graduate level.^16^, ^21^, ^22^, ^23^, ^24^, ^25^ This is especially relevant, as graduate students have been found to report high levels of academic‐related stress, at times even higher than that experienced by medical students or residents.^26^ However, the implementation of these assessment types in science, technology, engineering, and mathematics (STEM) courses has been under‐studied, likely due to higher reliance on the more traditional grading schemes used in STEM compared to what is often used in non‐science courses.^23^ Degree programs in these areas have also increasingly become modularized (combining multiple course units into one undergraduate degree), which requires that many topics be covered in a relatively short amount of time.^9^ Thus, there is little to no time available for formative assessment, and summative assessments predominate.^4^, ^10^ Ultimately, this minimizes opportunities for students to receive feedback that would greatly benefit their learning within challenging STEM disciplines.

Anatomy is one such discipline that has historically utilized predominantly summative approaches to grading—namely multiple‐choice or identification‐based (e.g., bell‐ringer) assessments, with some courses utilizing oral examinations or “*vivas*.”^27^ The use of multiple‐choice and identification‐based assessments has been critiqued in recent years, as they fail to evaluate integrated, higher‐level applications of anatomy and instead focus mainly on the lowest levels of Bloom's taxonomy (knowledge and understanding).^28^, ^29^ Oral *vivas* are commonly used in the training of medical professionals, such as clinicians or physical therapists, as they allow instructors to ask a greater range of questions while challenging students to incorporate anatomical and clinical terminology.^30^, ^31^, ^32^ However, due to the intensive role of the instructor in conducting oral *vivas*, these assessments are difficult to employ in large class sizes with outsized student‐to‐instructor ratios; hence, multiple‐choice and identification‐based assessments have remained the standard in most large anatomy courses.

This standard was recently challenged by the COVID‐19 pandemic, when students were forced into a remote learning environment that required most assessments to be delivered online. To deter student collusion (thus promoting academic integrity), many instructors changed their assessment approaches to focus on more formative assessments, such as lower‐weighted quizzes or reflection exercises.^24^, ^33^ This change was also found to enhance student motivation and engagement, as there was decreased reliance on less flexible quiz‐based, summative assessments, which typically offer less accurate representations of students' true learning.^31^, ^32^ Even before the emergence of the COVID‐19 pandemic in 2020, studies of student perceptions on formative assessments, such as written quizzes or unit examinations, had largely been positive;^33^ however, no such studies have explicitly investigated the impact of incorporating formative assessment into oral *vivas*.^33^

The Department of Anatomy and Cell Biology at Western University offers a graduate‐level anatomy course in which oral *vivas* are the sole method for assessment. This course is offered to graduate students in two course‐based master's programs, with students completing a full‐body dissection throughout four units, each taught by a different instructor. Historically, instructors in this course utilized a more summative, quiz‐based assessment approach, with set dates for oral *vivas* and questions that target specific concepts or details. In recent years, however, two of the four course instructors have incorporated a formative, feedback‐based, conversational approach to their assessment practice—a change that was made possible because of the small annual class size of about 15 students. This course, therefore, offers a unique opportunity to analyze the impact of de‐emphasizing grades using an alternative assessment approach in an anatomy course on graduate students' levels of assessment‐related anxiety when compared to more traditional assessment approaches.

Accordingly, the authors of this descriptive qualitative study investigated student and instructor perceptions related to feedback‐based assessment practices in graduate education. Specifically, the following questions were addressed through a constructivist approach to inductive thematic analyses of separate semi‐structured interviews with anatomy graduate students and instructors: (1) how do graduate students perceive the impact of assessment and grading in general on academic stress, motivation, and perceived quality of learning, (2) how do graduate students perceive the impact of the specific quiz‐based and feedback‐based formative oral *vivas* used in the course on their academic stress, motivation, and perceived quality of learning, and (3) how do the philosophies of the instructors on these matters align with the perceptions of their students? It was expected that the de‐emphasis of grades through feedback‐based assessment would help to alleviate students' assessment‐related anxiety, improve intrinsic motivation to learn, and lead to stronger perceived learning outcomes versus quiz‐based assessment approaches. The results of this study will help to fill gaps in the literature related to the use of formative assessment practices in graduate education, specifically in anatomy, to influence student stress and motivation.

## MATERIALS AND METHODS

### Course description

Participants were recruited from a graduate‐level anatomy course at Western University, which is open to students in two course‐based programs, the Master of Clinical Anatomy program and Master of Clinical Pathologists' Assistant program. As part of the course, all students participated in two‐hour seminars twice weekly (four hours per week) and two three‐hour laboratory sessions (six hours per week), the latter of which involved working in pairs or small groups to conduct a full‐body dissection over eight months (September 2022 to April 2023). Assessments came in the form of oral examinations, or *vivas*, and were distributed throughout the course. A total of 80% of the final grade was composed of their performances on four formative oral *vivas*—one for each of the four course blocks: Head and Neck (Block 1), Back and Thorax (Block 2), Musculoskeletal System (Block 3), and Abdomen and Pelvis (Block 4). So, the *viva* for each block comprised 20% of the final grade. The remaining 20% of the final grade was based on a final cumulative oral *viva* conducted at the end of the course (April 2023) that tested content from all four blocks using a traditional, quiz‐based assessment format.

Each of the four course blocks was taught by a different instructor and varied in duration: Block 1 was nine weeks, Block 2 was four weeks, Block 3 was five weeks, and Block 4 was eight weeks. Each instructor had a unique assessment style, which the study investigators have grouped into two categories based on similarities between them: quiz‐based assessments and feedback‐based assessments. Accordingly, Block 2 and Block 3 represented the quiz‐based assessment group, while Block 1 and Block 4 represented the feedback‐based assessment group. A summary of the course design is presented in Table 1, and a detailed breakdown of the assessment approaches used in each block is outlined in the following sections.

**TABLE 1:** Summary of course blocks, topics, and dates, as well as assessment styles, environments, and weights.

	Instructor's chosen assessment style	Block duration	Assessment environment	Evaluation weight (%)
Block 1 (head and neck)	Feedback‐based (formative)	September 12–November 10, 2022 (nine weeks)	Oral *vivas* in presence of laboratory partner	20
Block 2 (back and thorax)	Quiz‐based (formative)	November 14–December 20, 2022 (four weeks)	Oral *vivas* in presence of laboratory partner; 3 individual questions, 1 group question	20
Block 3 (musculoskeletal system)		January 9–February 16, 2023 (five weeks)	Oral *vivas* one‐on‐one with instructor	20
Block 4 (abdomen and pelvis)	Feedback‐based (formative)	February 16–April 14, 2023 (eight weeks)	Oral *vivas* in presence of laboratory partner	20
Final (cumulative)	Quiz‐based (summative)	April 26, 2023	Oral *viva* one‐on‐one with each instructor; 10 min with each instructor	20
			Total	100

*Note*: For the final cumulative *viva*, all instructors had predetermined questions and awarded students for logical attempts so that each question could be answered in individual ways by different students.

### Block 1 (head and neck)—Feedback‐based formative assessments

During this nine‐week block, students were assessed through regular conversations with the instructor using the body donor they dissected as a demonstration tool. During these conversations, the instructor would approach a table where a pair of students were dissecting and initiate a discussion on the students' objectives for the day (e.g., which branch of the facial nerve students were looking to identify in their dissection). The instructor might then ask students to point out relevant anatomical structures or ask them to elaborate on a structure's function or relationships to other structures. Students had the option to ask clarifying questions or to guide the conversation based on their unique progress. During these conversations, which could range in duration from approximately five to ten minutes, the instructor aimed to push students to the limits of their knowledge. Students were then provided with weekly written feedback on the course learning management system (LMS) after each assessment. The overarching feedback was provided as a descriptor term on a scale that included: “below expectations,” “acceptable as a pass,” “meeting expectations,” or “exceeding expectations.” During the first seminar of the block, the instructor made students aware that these descriptors were associated with a sliding scale that was created by one of the course instructors and used a complicated mathematical function to produce a numerical grade. Neither the students nor the instructors were able to directly discern how the function produced the associated grade, which was an intentional feature added to obscure the grading process and, thereby, redirect focus onto the assessment feedback. Students could view their numerical grades under the gradebook tab of the course LMS and visit a separate tab to view the feedback associated with each assessment. Each week was worth a varying percentage of the final 20%, ranging between 1% and 4%, with the percentage increasing throughout the nine weeks of the block. The percentage weight associated with each laboratory session was predetermined by the instructor and was consistent for all students.

### Block 2 (back and thorax)—Quiz‐based formative assessments

During this block, students were assessed through three oral quizzes using the body donor they dissected for demonstration. The three quizzes were spread across the four weeks of the block, with the third quiz being cumulative and covering content from all four weeks. In the first seminar of the course, the instructor acknowledged that their choice of this assessment style was mainly due to personal preference, but they did not elaborate further for students. Quizzes were conducted in small groups and lasted for about 15 min. The instructor asked each student individual questions from a set list, so each student received three different questions. A final question was then presented to the entire small group to be answered collaboratively. Students received numerical grades one to two days after the assessment, and generalized feedback was provided to the entire class either verbally or in writing on the course LMS.

### Block 3 (musculoskeletal system)—Quiz‐based formative assessments

During this block, students were assessed through two quizzes, each worth 10%, across the five weeks of the block. Quizzes were conducted one‐on‐one with the instructor and lasted between 5 and 10 min, during which the student was brought into a different room and was asked approximately three to five questions, depending on the amount of detail the student provided in their answers. Students were assessed using previously dissected specimens (prosections), rather than the body donor they were dissecting. The instructor provided no explanation to students regarding their decision to use their chosen evaluation style. After the assessments, the students would receive a numerical grade in their online gradebook without any explicit written or verbal feedback.

### Block 4 (abdomen and pelvis)—Feedback‐based formative assessments

During this eight‐week block, students were assessed through daily conversations with the instructor using the body donor they were dissecting as a reference. As with Block 1, the instructor would approach a table where a pair of students were dissecting and initiate a discussion regarding the students' objectives for the session. The instructor might ask students to point out relevant anatomical structures, have them elaborate on functions of structures, or ask them to describe relationships between structures. Alternatively, students had the option to indicate to the instructor one of 11 pre‐set competencies that they wanted to be assessed on for that laboratory session (see Supplemental Digital Appendix S1). Across these competencies, which included categories such as regional anatomy, embryology, comradery and collaboration, and other outcomes of interest, students were able to determine the weight that each would contribute toward their final grade. This approach incorporated elements of both standards‐based grading, which focuses on knowledge acquisition, and competency‐based grading, which focuses on the application of that knowledge.^34^, ^35^ Similar to Block 1, students also had the opportunity to ask clarifying questions during these conversations. After each conversation, the instructor would use voice‐to‐text to dictate individualized feedback that the students received and could view on the course LMS. Students also received rankings via the descriptor terms “below expectations,” “acceptable as a pass,” “meeting expectations,” or “exceeding expectations,” which were associated with the same sliding scale as used in Block 1 to produce numerical grades. However, the numerical grades for each laboratory session were not inputted into the students' gradebook, nor were they viewable on the course LMS. Students were only able to view a final grade at the end of the block. At the beginning of the block, students participated in a mandatory seminar in which the instructor provided a brief rationale for their chosen assessment style, as well as clarity regarding the 11 competencies and the structure of their conversational assessments. In this seminar, students were introduced to a progress bar, visible on their course LMS, which tracked their progression through the block based on their completion of the 11 competencies. A video summarizing the 11 competencies was made available on the course LMS.

### Participants

This study was approved by Western University's Non‐Medical Research Ethics Board (NMREB; project ID: 122238) in December 2023, before participant recruitment commenced. Students in the 2022–2023 cohort of the gross anatomy course (*n* = 15) took part in in‐person recruitment during the final seminar of the course in late April 2023, at which time informed consent was obtained via a Qualtrics survey (Qualtrics, Provo, UT) before participants engaged in semi‐structured interviews. This *n*‐value represents the total number of students in the cohort with the study investigator (ED) excluded. Instructor recruitment was conducted in September 2023 via email by a research assistant who was not affiliated with the course. The instructors (*n* = 4) also indicated informed consent through Qualtrics before participating in semi‐structured interviews. Demographic data were not reported for either group to maintain anonymity, given the small sample sizes. All data were stored within Microsoft Office Suite's OneDrive cloud service (Microsoft Corporation, Redmond, WA) on an institutional license through Western University using a participant ID code that was automatically generated by Qualtrics.

### Student interviews

A non‐validated semi‐structured student interview guide was created by the study investigators, aiming to elicit perceptions on how grading and assessment impacted students' academic stress, intrinsic motivation, and perceived quality of learning (see Supplemental Digital Appendix S2). Students were not limited to speaking about their graduate school experience, and many discussed their perceptions related to grading and assessment from their undergraduate education. The student interviews, which averaged about 45 min in length, were conducted on Zoom (Zoom Video Communications, San Jose, CA) by a study investigator who was a peer in the course (ED); this decision was intended to encourage transparent and candid responses from the participants and eliminate any power differentials that may have existed if the interviews were to be conducted by a professor. Furthermore, the interviewer's familiarity with the course, especially from the perspective of a student, allowed for deeper probing questions and greater insight into the nuances of the student experience. Audio recordings from all interviews were made using the Zoom record function and anonymized before transcription by the interviewer.

### Instructor interviews

A second non‐validated semi‐structured interview guide was created for the instructor interviews to determine whether the philosophies of the instructors aligned with the perceptions of their students, as related to the effectiveness of their chosen assessment practice and its effects on student anxiety, motivation, and quality of learning (see Supplemental Digital Appendix S3). The instructor interviews were conducted via Zoom by an external research assistant who was not affiliated with the course. The recordings were then anonymized and transcribed by a research team member (ED).

### Data familiarization and analysis

All audio recordings were transcribed by a study investigator (ED) into Microsoft Word. Separate inductive thematic analyses were then performed on the students' and instructors' transcripts to draw semantic themes from their interview responses using ATLAS.ti software, version 23 (ATLAS.ti Scientific Software Development GmbH, Berlin, Germany). A constructivist approach to analysis was utilized, with an awareness of the students' unique personal experiences and social constructs present in the course; namely, the assessment environments created by each of the four instructors.^36^ As such, this approach was aimed at providing the most comprehensive, multi‐dimensional evaluation of the influence of the varying assessment practices.

The following six phases of thematic analysis, as originally outlined by Braun and Clarke (2006), were used in an iterative and reflexive process: (1) data familiarization, (2) formulation of initial codes from the data, (3) generation of preliminary themes based on relevant codes, (4) revision of themes through repeated interaction with the data, (5) definition of themes, and (6) production of the final manuscript.^37^ The transcription process and repeated readings were used as the first phases of early data familiarization and analysis, then initial codes were formulated and organized within the ATLAS.ti software. Generation of the preliminary themes and subthemes was achieved through the creation of unique participant “profiles,” in which mind map visualizations that sorted and linked related codes were made for each participant using the Canva online graphic design software (Canva Inc., Sydney, Australia). An example of one such profile is available as Supplemental Digital Appendix S4. These profiles were then compared to one another to identify similar themes across interviews from the same population (students or instructors). This process further promoted the authors' familiarization with the data, as an element of the recursive process of thematic analysis. All coding was conducted by the first author (ED), but regular discussions were held with the senior author (SM) to refine the codes and subsequent themes. The first author was mindful of their unique position as a student in the course, both when interviewing fellow students and when coding interview transcripts and integrated that identity into the analyses by engaging in reflexive exercises such as personal reflections on positionality. Positionality and reflexivity were also discussed in the regular meetings between research team members throughout the analytical process to promote awareness of the lens through which the data were being assessed. This approach is in line with Braun and Clarke's most recent description of *reflexive* thematic analysis, which utilizes the researcher as a central instrument for data analysis and acknowledges their inherent subjectivity as a strength of the process.^38^

## RESULTS

### Student data

In total, eight of the nine (89%) Clinical Anatomy students and one of the six (17%) Clinical Pathologist's Assistant students consented to participate in the study, for a total of nine (60%) student participants in the interview process. The analysis of the interview transcripts generated the following semantic themes related to alternative assessment and grading, as summarized in Table 2: (1) communicating clear goals and expectations, (2) instructor approachability and individualized assessment, and (3) alignment between perceived effort and outcomes.

**TABLE 2:** Themes and subthemes generated through a constructivist approach to thematic analysis of semi‐structured student interviews (*n* = 9). Example quotations for each subtheme are provided.

Theme	Subtheme	Related quotation
Communicating clear goals and expectations	Unclear instructor intentions	“…[the instructors] may have told us how they grade, but it's hard when you don't know [what] the expectations are”
	Higher expectations in graduate education	“…program standards [are] already high … we had to get 80% in everything to pass every course, so I think there's always some apprehension around grades”
	Reassurance from instructors	“…it's definitely a lot more stressful when you have no idea, no notion of how you're doing”
Instructor approachability and individualized assessment	Perceived instructor investment	“…the [instructors] that were very supportive in your learning [made] a big difference [with anxiety]”
	Flexibility at the graduate level	“…I think there's less rigidity in terms of learning with graduate versus undergraduate anatomy courses”
	Receiving feedback	“Based on how much I enjoyed [the] feedback, I think it played a role to engage in [fostering] a deeper level of learning”
Alignment between perceived effort and outcomes	Disconnect with feedback	“I kept getting ‘meeting expectations’ and not exceeding, like, what am I doing wrong? I'm trying my best”
	Disconnect with grades	“I don't think that my grades necessarily reflect my level of learning in [traditional blocks]”
	Misalignment with evaluation style	“…with bell‐ringers, you might know the content, but you just don't understand what [instructors] are asking for”

### Communicating clear goals and expectations

In general, the students all believed that one of the biggest influences on their academic‐related anxiety and motivation to learn in the course was the level of clarity that instructors provided when describing their goals for and expectations of the students. Many students highlighted the impact of unclear instructor expectations on their experience, while others also pointed to the idea that expectations of graduate students were inherently higher than those of undergraduate students. They also indicated a need for some form of reassurance from their instructors to ensure that they felt as though they were satisfying those expectations. These experiences were felt consistently across the blocks despite their various instructors and assessment styles.

### Unclear instructor intentions and higher expectations in graduate education

Students expressed that graduate education placed higher expectations on their understanding and application of material, specifically in the discipline of anatomy, when compared to the undergraduate level. They felt that clear communication of these goals and expectations by all instructors was essential, not only for their learning outcomes but also to reduce their academic‐related stress and the anxiety caused by uncertainty regarding expectations, as one student described in relation to their experience in a quiz‐based block: “*It made me a little more stressed throughout because I didn't know what was expected of me, like I had to fill in those gaps for myself*.” Other students described feeling enhanced motivation to remain on top of the content when they felt like they understood the expectations of the instructor, with one student reporting:

[the instructor's] questions reflected what I thought we were supposed to be taking away from the block … and so, I felt pretty motivated to keep up with the material because I was like ‘OK, like everything that I'm learning here could be applicable, and even if it doesn't get asked on the viva, I know that it's still important to know’.

In contrast, students reported that instructors who utilized feedback‐based formative assessments typically provided more clarity surrounding their goals and expectations for students.

### Reassurance from instructors

Students also discussed their reliance on feedback and encouragement to determine whether they were meeting the expectations of the instructors, or at least on the right track, especially when the expectations were unclear. They described a need to interpret body language cues from their instructors during the evaluation and indicated heightened stress associated with instances when they were unable to discern their performance, as one student highlighted from an instructor in a quiz‐based block: “*It is a bit stressful because [the instructor] didn't give any sort of response, and it kind of makes you panic a bit more*.” Some students reported relying on their peers “*for reassurance*” to supplement what they felt was lacking from the instructors, especially in blocks where they were assessed in front of their peers or when they had “*no notion of how [they] were doing*”, based on the stoicism of the instructor. However, others acknowledged the potential benefit of instructors remaining stoic during the evaluation, stating, “*It was helpful in the sense that you're just gonna say [an answer] and you're going to have to stick to it, because [the instructor] is not going to tell you, so you'll just have to go for it*.”

### Instructor approachability and individualized assessment

Students in this study described how their levels of motivation and stress were influenced by their perceptions of instructor investment in their learning and the degree of flexibility provided in assessments at the graduate level. They highlighted that instructor approachability impacted their willingness to seek feedback and that the level of individualization provided in this feedback could either benefit or hinder their perceived learning experience. Students demonstrated a strong preference for individualized feedback from their instructors, which helped them direct their learning and understand their performance more meaningfully.

### Perceived instructor investment

In general, students described feeling changes in their motivation to learn depending on whether they perceived that the instructor had an investment in their learning. One student described experiencing variations in their stress levels depending on the perceived investment of the instructors. Instructor investment also played a role in how approachable they were perceived to be by the students, which influenced how they interpreted the feedback provided by the instructor. One student described having a more challenging time asking for additional feedback from an instructor whom they viewed as less approachable, which resulted in increased stress: “*Asking for feedback kind of [felt] like you're failing because you didn't meet [their] expectations once*.” This student then went on to explain how that experience influenced their motivation:

I feel like if you already had a really negative experience in the evaluation, it's hard [not to] read feedback that might be positive in a way [without it] having some tinge of negativity … based on that situation, I don't think I would be able to perceive a lot of what [the instructor] would say as a motivator for my future studies.

### Flexibility at the graduate level

Students discussed a preference for the flexibility that existed within their assessments at the graduate level, especially when compared to the bell‐ringer examination styles that are commonly used at the undergraduate level. One student highlighted that the use of individualized oral assessments allowed them to “*interact with [their] thoughts and work [answers] out with [instructors]*,” and that this approach helped to enhance their learning versus the rote memorization approaches they utilized for bell‐ringer examinations. The individualization present in the course also gave students the ability to highlight their knowledge and be rewarded for it, rather than penalized for what they did not know. This helped to alleviate students' stress, as one student described when comparing their experience in the course to their undergraduate education:

…[some instructor's questions] did not have just one correct answer, so we could have a conversation that could drift into whichever direction we wanted…I really liked that [the instructors] did not focus on what we did not know, whereas in undergraduate [teaching assistants] would find out that you ‘don't know’ and just keep dragging it on and on, so you get a zero out of five.

### Receiving feedback

Students demonstrated a preference for both individualized assessment and feedback, which they perceived to offer benefits related to their motivation and academic‐related stress. For example, one student highlighted that the individualized feedback they received “*helped my motivation, because then I knew what I should be focusing on rather than just a broad statement*.” Others felt that the specificity of the feedback made it more meaningful for their learning:

I really liked the individualized feedback as opposed to [the generic feedback] that doesn't really mean anything to me. If my name is in the [feedback] and [it] really points out a specific thing that I did, it feels so much more usable.

Compared to the quiz‐based approach, students found that the conversational style of the feedback‐based approach allowed them to demonstrate their knowledge without being penalized for feeling less confident with other topics. They also indicated a preference for the order in which they accessed their feedback and grades, stating that they viewed their grades more favorably when they read the feedback first and thus preferred the feedback‐based block wherein feedback was provided on a separate tab of the LMS from the grades (Block 1).

### Alignment between perceived effort and outcomes

Students in this study discussed experiencing various disconnects and misalignments in their educational experiences, both in this course and in general. At times, they felt that the effort they invested in preparing for an assessment did not align with the corresponding feedback or grades they received, which led to enhanced stress and a questioning of their performance. Similarly, students expressed concerns regarding the alignment of assessment styles with learning outcomes, which influenced their stress and motivation levels.

### Disconnect with feedback and grades

Overall, students reported that they occasionally experienced disconnects between the amount of effort they put into their preparation for assessments and the amount of feedback or resulting grades they received. One student recounted how the feedback they received in one of the feedback‐based blocks negatively impacted their stress levels: “*It added to my stress because when I kept getting, like, ‘meeting expectations,’ and not ‘exceeding,’ I was thinking ‘what am I doing wrong,’ like, I'm trying my best*.” Others echoed the feeling that they, at times, experienced a misalignment between their perception of how the assessment went and their subsequent grade, which impacted both their stress and their motivation toward future assessments. One student explicitly discussed the influence that grades had on their motivation, in general:

It [is] very demoralizing when [the grades] don't work out and then, in that case, it's not a good motivator because you feel like the work that you're putting in isn't getting you that reward that you thought it was.

Students also highlighted experiencing some disconnects with the amount of feedback they received in feedback‐based blocks, compared to what they believed the instructors were going to provide them, indicating that when feedback was less detailed and specific than they expected, *it* “*kept me kind of unmotivated*.”

### Misalignment with evaluation style

Students also touched on misalignments or disconnects between their efforts, the outcomes, and the instructors' expectations, as related to the style of the assessment. One student discussed a misalignment they experienced in a quiz‐based block, stating:

When the assessment doesn't really reflect what's being taught or the expectations of what you should be getting out of what's being taught, it's like ‘why am I doing this?’

Students described experiencing this misalignment both with the oral *viva* evaluations used in this course, which they likened to bell‐ringer assessments that many had previously participated in during their undergraduate anatomy education, stating, “*… with bell‐ringers, you might know the content, but you don't understand what [instructors] are asking for*.” From this perspective, students stated that they preferred having oral evaluations, as they could seek in‐the‐moment clarification from instructors.

### Instructor data

All four instructors (100%) who shared instructional duties in the course consented to being interviewed. Through inductive thematic analysis, three main themes related to instructors' intentions were identified, as outlined in Table 3: (1) promoting non‐technical, discipline‐independent skills (NTDIS), (2) providing authentic learning experiences, and (3) emphasizing individualized assessment.

**TABLE 3:** Themes and subthemes generated through a constructivist approach to thematic analysis of semi‐structured instructor interviews (*n* = 4). Example quotations for each subtheme are provided.

Theme	Subtheme	Related quotation
Promoting non‐technical discipline‐independent skills	Autonomy and decision‐making	“I want [students] to feel they can put emphasis on things they value, considering their future goals”
	Communication	“Knowing most of these students will become [teaching assistants], it's not just about their knowledge, but their teaching skills”
	Empathy and humility	“…what I can do is make [students] into better, more well‐rounded people”
Providing authentic learning experiences	Oral evaluations	“…it's more real‐world applicability to think on your feet”
	Public and frequent evaluations	“…it's meant to be emphasizing real‐world skill sets, like being able to communicate to your peers”
	Providing constructive feedback	“…in the real world you're assessed all the time, so when you have a bad day, it shows but doesn't have to be your undoing”
Emphasizing individualized assessment	Guiding conversations and addressing gaps in the moment	“I'll try to redirect [students] during their conversation, if they're going too broad or too deep”
	Student confidence and stress	“I can tell, [students] walk in and they're almost shaking, so I try to disarm that, just get the nerves calmed down”
	Logistics of individualized assessment	“because of [student] numbers…I think we can…embrace more personalized opportunities [at the graduate level”

### Promoting non‐technical, discipline‐independent skills (NTDIS)

In general, the instructors all believed that their role went beyond merely teaching proper dissection‐ and laboratory‐based skills. When describing their overall goals for students, each instructor highlighted the desire to cultivate the development of transferable skills as a central tenant of their teaching philosophy. These skills, which have at times been described as “*soft skills*,” were deemed important by individual instructors based on their own experiences as students and later as instructors, which they had cultivated through conversations with other instructors and professionals. Typically, the instructors focused on addressing skills that they felt had been traditionally lacking after students finished their academic program.

### Autonomy and decision‐making

Instructors acknowledged that, despite this group of students participating as teaching assistants during their academic program, not all of them would go on to careers in instructional settings. As such, many described wanting to support the development of the students' autonomy and decision‐making within their education. For example, an instructor who utilized a feedback‐ and competency‐based approach to assessment stated that they were “*trying to empower the students*” by allowing them to individualize the weightings of each competency that was assessed, which required the students to think critically about what areas mattered most to them. This instructor described satisfaction in having students provide rationales for the weightings they selected, stating:

I want [the students] to feel like they can put emphasis on the things that they value the most, considering their future goals and considering their next steps. I want that rationale so that, again, they're thinking about where these competencies are fitting, and it's larger than just the time they're with me.

### Communication

One instructor explained their decision to place emphasis on students' abilities to go on to teach the material in future instructional roles:

My philosophy in teaching is, while I think it's great that [they] know anatomy, they're going on to teach it. So, you can look it up in a book, what's there, anybody can do that. How you are approaching it—that's the real skill that I want them to have at the end.

Another instructor echoed the same sentiment: “*Knowing that most of these students will become important [teaching assistants] in the subsequent year, it's not just about their knowledge but their teaching skills that I try to improve*.” The more specific elements of teaching that were discussed by instructors included enhancing students' abilities to clearly explain diverse topics, encouraging summarization and synthesis of relevant information, and developing students' confidence in their delivery of material. Instructors also believed that the use of oral assessments “re*quires [students] to think on their feet*,” which they described as an important skill for students to develop as teaching assistants who will inevitably be approached with questions that are novel to them.

### Empathy and humility

In a teaching role, where students are asking novel questions, the instructors felt that the graduate students should be expected to learn how to be able to admit that they might not always have the correct answer. This was seen as especially relevant for young teaching assistants, who might feel pressured to know everything and never make mistakes. One instructor highlighted the importance of humility and self‐awareness in these moments, stating: “*As a teacher, humility is not a bad thing, it's not a weakness, it certainly can be perceived as a strength*.” They believed it was part of their role as an educator to foster a sense of humility in their students and help shape the person they become by the end of their academic program. As such, one instructor expressed their preference for having one‐on‐one interactions with students, as they believed it made students more comfortable with acknowledging the instances where they make errors or misspeak. Many of the instructors shared a common goal of having their students leave their block as “*better people*,” with one instructor saying:

My philosophy is that I'm not there to teach you content because Google is pretty good at that and I'm probably never going to be as good as Google, but what I can do for them is make them into better, more well‐rounded people.

### Providing authentic learning experiences

In general, the instructors highlighted the requirement of their evaluations to prepare students for their future careers, which was often equated to “real‐world applicability.” As such, all of the instructors discussed their preference for an oral style of evaluation that occurred in a public setting at frequent intervals to promote development of public speaking skills. They also outlined their perceptions regarding the provision of constructive, individualized feedback to students.

### Oral evaluations

Overall, instructors expressed that their approach to assessment was focused on applicability to the “*real world*.” This included the exclusive use of oral assessments in the course:

I actually really like the fact that it's an oral exam because … it requires them to think on their feet [and] I think it's more real‐world applicability to think on your feet and try to answer the question using or applying the knowledge that you do have.

This “thinking‐on‐your‐feet” approach required students to be adaptable and sufficiently well‐versed in the material, which one instructor described as important since real‐world scenarios do not follow the same format of offering “*a chance to write an essay … or to see the answers [as part of] multiple choice [questions]*.” Instructors also highlighted that the nature of these assessments allowed them to base their questions on the responses of the students, enabling the assessment to be individualized to highlight each student's strengths and areas for improvement.

### Public and frequent evaluations

In addition to the perceived benefits of oral assessments, some instructors believed that having frequent public evaluations, where students were being evaluated in front of their peers, was useful for helping them to further develop NTDIS:

I opted for things like a public evaluation, and it's meant to be emphasizing and engaging those real‐world skill sets, like being able to public speak [and] being able to communicate in front of your peers.

The frequency of evaluations was also thought to prepare students for the types of evaluations that they might experience outside of their academic program since, as one instructor described, “*in the real world you're not just judged sometimes, you're judged all the time when you're in the work environment*.”

### Providing constructive feedback

According to many of the instructors, individualized feedback on work will often accompany the frequent evaluations students will likely face in real‐world scenarios; thus, students need to learn to be receptive to frequent feedback and critique and able to apply that to future work. One instructor highlighted the importance of feedback as an iterative process that also de‐emphasizes the students' focus on their grades:

In the real world, we recognize that students rarely have an opportunity to continue working on something after they've submitted it for grading. So, my philosophy here was emphasizing the idea that just because you got a good grade on it, doesn't mean it's done, it doesn't mean you can't keep working on it.

With the frequent reception of feedback, students are encouraged to continue learning and striving to improve, which is a skill that instructors believed they will need when working both within and outside of academia. They indicated that students will also need to learn to receive constructive yet critical feedback in the workplace, which was the intention behind the assessment style of one of the instructors:

Most feedback is largely critical, hopefully constructively so, and this idea of getting used to like ‘Oh, I didn't quite hit it this week’ is really important. So, trying to get them used to, you're going to have good days, you're going to have bad days in the real world, you're assessed all the time so when you have a bad day it shows, but it doesn't have to be your undoing.

### Emphasizing individualized assessment

When speaking about their assessment approaches, instructors cited their emphases on providing students with individualized education. Throughout the instructor interviews, thoughts on guiding conversations, helping students develop confidence, and addressing knowledge gaps in the moment were common, along with logistical considerations and potential limitations of implementing individualized assessments.

### Guiding conversations and addressing gaps in the moment

During the assessments, instructors highlighted their ability to tailor the conversational assessments based on the responses provided by students, as one instructor explained: “*As the student sort of starts to answer the question, the follow up might be a little bit different depending [on their answer]*.” This sentiment was even shared in the context of the quiz‐based oral assessments, where instructors discussed that students could be rewarded for any “*effort that actually is logical, based on what they know about anatomy*,” rather than being penalized for not arriving at a specific conclusion or definitive answer. Not only did this individualization allow students to determine the direction of their assessments, but it also benefitted students who needed additional guidance, as the instructors felt able to redirect the conversation. For instance, one instructor described: “*I'll try to redirect them during their conversation if they're going too broad or too deep*,” at which point they could challenge the student to add additional detail or reiterate key topics. These instances also allowed the instructors to address specific gaps in a student's knowledge in the moment, rather than waiting until a formal or summative assessment to determine and highlight those areas.

### Student confidence and stress

Instructors also perceived that their focus on individualized assessment in the course helped them to support students in addressing potentially lacking confidence with the material. For example, one instructor described a preference for having one‐on‐one conversational assessments with students because they “*allow for people to really show their abilities, or if they have [a] lack of abilities or confidence, then we can have a more personal discussion about it*,” while mitigating the potential for students to feel embarrassed about having those conversations in front of their peers. Using an individualized approach also helped the instructor to minimize the assessment‐related stress experienced by students by making initial evaluations of the student's mindset and approaching the assessment accordingly. One instructor described their experience with facilitating student's stress during their assessments accordingly:

I can tell, [students] walk in and they're almost shaking, I try to disarm that, I try to start with kind of an easy question just to get the conversation going, just to get the nerves calmed down.

By taking this tailored approach to address students' stress, instructors were able to guide the assessments in ways that allowed students the chance to maximally demonstrate their knowledge.

### Logistics of individualized assessment

While every instructor discussed the benefits of emphasizing individualized education for their students, they also acknowledged the logistical limitations, along with some considerations for implementing individualized assessment. The largest limitation discussed was class size: instructors each highlighted that the time and resources required for individualized assessment precluded it from being used in large undergraduate‐level classes. One instructor explained how their approach would only be effective at the graduate level: “*I think that's a strong difference, between the necessity of having more of a cookie‐cutter approach at the [undergraduate] level just because of [student] numbers, and I think we can let that go and embrace more personalized opportunities [at the graduate level]*.” Multiple instructors acknowledged that an individualized approach is often avoided at the undergraduate level for fears of assessments being perceived as unfair, and the same concern was raised in the context of this course. One instructor who utilized the quiz‐based assessment approach described their decision to incorporate set questions “*so there's no chance of one student feeling like they didn't get their fair shake… and then they just happened to be on a day that was much more difficult for them*.”

## DISCUSSION

Alternative assessment practices, such as feedback‐based assessment, have grown in popularity in an attempt to move students away from a grade‐focused mindset and the associated academic‐related stress toward more intrinsically driven motivations to learn. Applications of these practices are still being explored, both across disciplines and across different levels of education. To further understanding of the impacts of these such approaches, the authors of this study sought to investigate the perceptions of both students and instructors on the use of feedback‐based versus quiz‐based assessment in a formative evaluation setting as part of graduate anatomy education. Interviews were conducted to explore students' perceptions on assessment and grading, both in general and as related to feedback‐based assessment. They were then analyzed through inductive thematic analysis to yield the following semantic themes: communicating clear goals and expectations, alignment between perceived effort and outcomes, and instructor approachability and individualized assessment. Instructor interviews analyzed using the same approach described an overlapping theme of emphasizing individualized assessment, as well as distinct themes related to promoting NTDIS and providing authentic learning experiences. When examining the themes from the instructor and student interviews together, the frameworks of constructive alignment and teaching presence most accurately fit the experiences described by all participants. Accordingly, these two elements will be discussed within the context of the feedback‐based and quiz‐based formative assessments used in this course.

### Successes and failures in constructive alignment

A common experience among students in this course was a feeling of uncertainty surrounding the expectations and goals of their instructors. During the course blocks that utilized feedback‐based assessments, these feelings of uncertainty were often enhanced, even if only briefly, despite instructors believing that they had adequately explained the philosophies behind their chosen assessment style to the students. For example, some students reported feeling confused as to why they were sometimes evaluated in front of their peers, whereas other times they were evaluated individually, depending on the instructor. This was reported despite a consensual understanding among students of why all four of their instructors chose to use oral evaluations.

This disconnect emphasizes the importance of considering constructive alignment in the implementation of any evaluation, especially when exploring novel or alternative assessment approaches. This concept, which combines constructivist theories of learning with instructional design, encourages educators to structure both their teaching activities and evaluations to match the predetermined learning objectives of their course, in order to support students as they construct meaning and develop knowledge during the learning process.^39^ To fit the constructivist lens, the learning objectives, activities, and assessments must also be authentic and relevant to the context in which the students will use their knowledge in the future.^40^ Studies that investigated the influence of constructive alignment in higher education established that utilizing constructively aligned methodologies predisposed students to employ deeper approaches to learning and enhanced their intrinsic motivation.^41^, ^42^

In the present study, students reported feeling more motivated to stay on top of the content if and when they perceived that there was appropriate alignment between their learning outcomes and the instructor's chosen assessment style, regardless of whether that was a feedback‐based or quiz‐based assessment. This mirrors the aforementioned findings in the literature.^41^, ^42^ Conversely, when alignment was perceived to be lacking, students felt increased academic‐related stress (Table 2). Stamov‐Roßnagel and colleagues (2021) reported this same increase in students' stress when they experienced misalignment between learning objectives and the style of their evaluation, which contrasted with feelings of less academic‐related stress and heightened competence among students who experienced proper alignment.^43^ Taken in the context of the existing literature, the present findings, therefore, support the importance of forethought in designing constructively aligned courses, especially when introducing non‐traditional assessment approaches.

### Hidden curriculum: Non‐technical discipline‐independent skills (NTDIS)

Also related to constructive alignment, one of the largest potential factors that can contribute to the academic‐related stress experienced by students is the failure of instructors to adequately articulate their teaching goals and expectations. This is especially relevant in relation to the instructors' hidden curricula, or the implicit teachings the instructors attempt to instill in their students.^44^ Each of the instructors in this study described intentions to not only teach their students anatomy‐related skills, such as proper dissection techniques and spatial understanding, but also a myriad of NTDIS that have translational, “real‐world” applicability. Common desirable NTDIS suggested in the literature include communication, collaboration, autonomy, and empathy, as well as NTDIS that are specific to anatomy, such as respecting social norms in the laboratory and navigating the cultural and ethical implications of body donation and cadaveric dissection.^45^ Indeed, these were emphasized by the instructors in this study (Table 3).

Overall, NTDIS have been well‐established as elements that should be integrated into curricula alongside content‐related outcomes to best prepare students for their future careers within and beyond academia.^46^ Some students in the present study claimed to understand their instructors' intentions clearly, both related to the practice of technical skills and the development of NTDIS. For example, despite some instructors preferring to examine students in front of their peers versus individually, the students suggested that they generally understood that the instructor was doing so to target communication skills through public evaluations. However, there were also opposite cases in which students felt uncertain of the instructors' assessment‐related decisions. For instance, some students expressed confusion as to why they would be separated from their partner for an individual assessment in one block after previously being evaluated as a group in others. This disconnect reportedly increased their anxiety related to the assessment.

Related to that disconnect, students also felt that NTDIS were not adequately highlighted by the feedback they received in any of the blocks. In contrast, the instructor interviews emphasized a focus on promoting NTDIS as a goal of their teaching and assessment practices, indicating a clear divide between the instructors' intentions and the students' lived experiences. The numerous challenges related to cultivating NTDIS in gross anatomy courses, including barriers such as reduced laboratory contact hours, are well‐described in the literature.^45^, ^47^ Interestingly, despite their best intentions, similar barriers were acknowledged by the instructors in this study, indicating that not only are the instructors faced with logistical difficulties that limit their ability to promote NTDIS, but that an equal or greater barrier to the effectiveness of their efforts may be a failure to clearly communicate their intentions to their students. Accordingly, regardless of the assessment approach, instructors should carefully and intentionally communicate their goals for their students' learning and describe how their teaching and assessment practices align with those intentions.

### Emphasizing NTDIS using alternative assessment and grading approaches

Considering these and other challenges, instructors in this study have made strides toward ingraining NTDIS into their block curricula. For example, in blocks that utilized quiz‐based assessment, instructors relied heavily on the use of oral evaluations alone to allow students to develop their communication and teaching skills. One might plausibly argue that these blocks also challenged skills such as problem solving and, in some cases, teamwork as students worked through identification and application questions related to the anatomy. However, one of the instructors that utilized feedback‐based assessments attempted to further enhance these such outcomes by incorporating elements of both standards‐ and competency‐based alternative grading practices into the oral assessments to de‐emphasize grade‐based motivations to learn and target NTDIS development through intrinsic motivation.

Standards‐based grading (SBG) evaluates students' success at demonstrating mastery of the outlined learning objectives, or “standards”; whereas competency‐based grading (CBG) is the measurement of students' mastery of previously outlined competencies, which align with how students are intended to apply their knowledge within novel and authentic contexts.^34^, ^35^ Plainly, SBG can be thought of as focusing on a student's knowledge, while CBG assesses what they do with that knowledge. Both SBG and CBG have been demonstrated to reduce students' stress in undergraduate settings.^34^, ^48^ Additionally, CBG has been associated with the promotion of deeper learning, which is a factor of intrinsic motivation and the use of deep learning strategies.^34^ Thus, the implementation of SBG and CBG by an instructor in this course may have been one contributing factor to students' decreased academic‐related stress and enhanced intrinsic motivation reported during that block. In addition to student‐centered *assessment* practices such as feedback‐based assessment, SBG, CBG, and other such approaches to *grading* could, therefore, offer an avenue to provide more meaningful course experiences for students. Instructors who are interested in exploring strategies related to alternative grading should examine literature and resources on “ungrading”.^49^, ^50^

### Individualized assessment and high‐quality feedback

With a small cohort of students, instructors have greater freedom to implement novel strategies when targeting NTDIS development, and they are better able to tailor their assessments to target each student's individual areas for improvement. Use of individualized formative assessments in this manner has been demonstrated to enhance academic performance, as well as mitigate test‐related stress and anxiety.^51^, ^52^ While this study did not investigate numeric performance or any other quantitative metrics, many students reported that having an individualized, self‐directed approach to assessment resulted in feelings of enhanced intrinsic motivation because they had the freedom to guide their assessments toward areas that better highlighted their own unique knowledge. They also indicated a reduction in the stress that they experienced related to those assessments, which echoed findings in published literature linking self‐directed learning to enhanced resilience.^53^

An element of individualized assessment that was discussed by both students and instructors in this study was the provision of feedback following formative evaluations. Effective feedback is a core component of high‐quality learning and is characterized as the provision of specific, actionable, objective, and non‐evaluative commentary on student performance that is delivered promptly, though the type and timing of delivery can vary.^54^ Students in this study demonstrated a preference for receiving written, individualized feedback (associated primarily with the blocks that featured feedback‐based assessment), noting that since they had documentation of the feedback they received, they could better monitor their progress and direct their learning in response. They also indicated a preference for rapid receipt of feedback so that they were sure it was up‐to‐date, meaningful, and relevant to what they had been working on in recent laboratory sessions. These were noted by Gibbs and Simpson (2005) as important features of high‐quality feedback and should be implemented by instructors striving for the provision of valuable formative assessment.^10^

The order in which students received the feedback was another, less anticipated factor influencing their perceptions. One of the instructors who used feedback‐based assessments provided students with both written feedback and the associated numeric grade within the same tab on the LMS, while the other instructor provided the feedback in a dedicated tab that was separate from the final numeric grade. While the instructors did not indicate an explicit rationale for why they chose their given method of feedback presentation, students were more appreciative of having the feedback separated, as it allowed them to reduce their focus on the numeric grade. Over time, many found themselves to have gradually shifted to preferentially seek out the feedback rather than the grade, demonstrating the more learning‐focused mindset that their instructors had originally intended to foster. This enthusiasm for receiving feedback became so great that some students reported feeling a “*sting*” of disappointment when they did not receive the amount of feedback they expected. This was an example of another area of disconnect between students and instructors: most instructors believed that they were consistently providing adequate feedback to their students, while most students reported to have received what they felt to be sufficient and useful feedback in only one block. Clearer communication from the instructors to indicate the amount of feedback that they intended to provide could, therefore, allow students to adjust their expectations when seeking and receiving feedback.

### Role of teaching presence and implications for cognitive load

How students perceive the feedback they receive can be influenced by the environment created by their instructors. This is an element of teaching presence, which, along with cognitive presence and social presence, contributes to fostering a community of inquiry that serves to support learning through the construction of knowledge in a collaborative environment.^55^ Cognitive presence refers to opportunities for the development of knowledge through reflection and critical analysis, while social presence describes the degree of success at creating a supportive environment in which learning occurs.^56^ An instructor's teaching presence guides the cognitive and social presence of their learners through course design, facilitation, and instruction, and has been correlated with enhanced student engagement and motivation when well‐achieved.^57^, ^58^

This was supported in our current study, as students reported decreases in their motivation when instructional interactions were fewer. This, in turn, caused increases in students' stress in anticipation of the next interaction. Facilitation of a judgment‐free learning environment was also highlighted by students as a primary role of the instructor in minimizing students' stress and encouraging them to actively engage in the learning process. This effect is thought to be moderated in part by the attenuation of cognitive load through instructional course design, as described in relation to student motivation by Zhang and colleagues (2023).^59^ The experiences of stress in the present study varied between students, as well as between blocks, due in part to variations in block length and the amount of time spent with each instructor. Notably, this influence was not found to be directly associated with a particular assessment style, but rather with the instructor themselves. A variety of sociocultural or demographic factors can contribute to this influence, resulting in impacts to both teaching presence and students' social presence. For example, instructor gender has been reported to influence stress and academic performance for some students, with female instructors positively influencing these outcomes among female students, which may be due to a role‐model effect.^60^, ^61^

Regardless of sociocultural factors, implementing ways of teaching or assessment that universally reduce extraneous cognitive load and optimize the demands on students' working memory should be of major importance to instructors due to the established connection between cognitive load, memory, and recall.^62^ This is especially relevant for assessments in anatomy, as it is a discipline that is associated with a high intrinsic cognitive load based on the quantity of information being learned.^63^ For many students, an assessment environment of any kind is inherently stress‐inducing. While this can be partly mitigated by an instructor's teaching presence, any experience of elevated stress levels can interfere with working memory and have impacts on performance.^64^ Students in this study reported that they experienced varying levels of stress between quiz‐based and feedback‐based assessments and that the reduced stress experienced during the smaller, feedback‐based assessments allowed them to retain more of the information being presented. This indicates that the more flexible, feedback‐based assessments may offer one strategy for reducing students' academic‐related stress in graduate anatomy education.

### Benefits and drawbacks of feedback‐based formative assessment

The potential for stress reduction, thus minimizing the impact on cognitive load and memory recall, is just one example of a benefit of incorporating feedback‐based assessments into a formative anatomy evaluation setting. Students' stress can also be decreased through the enhancement of intrinsic motivation, a connection that has been previously demonstrated by post‐secondary education research.^65^ Students in the present study described feeling both increased intrinsic motivation and subsequent decreased stress in blocks utilizing feedback‐based assessments. This intrinsic motivation was fostered, in part, by the provision of individualized, detailed feedback, which helped students to refocus toward a learning‐centered mindset. This mindset was also adopted by the instructors for those blocks, who explained that their implementation of feedback‐based assessment practices helped them to minimize the subjectivity of assigning seemingly arbitrary percentages to student performance. They also noted that providing feedback to students and witnessing their subsequent growth and improvement resulted in personal satisfaction with their teaching, which aligns with evidence from other research.^54^

Despite the benefits to instructors and students alike, feedback‐based approaches involve logistical considerations which can make their implementation complex. For example, instructors face the burden of providing detailed feedback to each student, while ensuring that it is delivered in a short enough timeframe for the feedback to be effective.^66^ Additionally, since the utilization of multiple, smaller evaluations requires the instructor to interact with every student in the form of assessment, time for teaching and facilitating learning is forced to be redistributed, which can leave some students feeling ignored or perceiving their instructor as being uninvested in their learning, which can have negative implications for student engagement.^67^ In contrast, the quiz‐based assessment approach, which included fewer evaluations and minimal feedback, was logistically simpler for instructors and granted them the ability to circulate more freely for teaching interactions during the laboratory sessions. One instructor also found it less demanding to prepare a set list of questions in advance; plus, they perceived the use of the list to be fairer to students, as each would receive the same balance of questions with varying difficulties. While this may aid in mitigating instructor burnout and normalizing laboratory assessments, it may lack the benefits to students that have been shown to be associated with more individualized assessments.^66^, ^68^ Instructors must, therefore, balance logistical considerations related to class size and contact hours with their efforts to provide an individualized and transformative learning experience.

### Considerations for implementation of feedback‐based formative assessment: FOCUS

**FIGURE 1:**
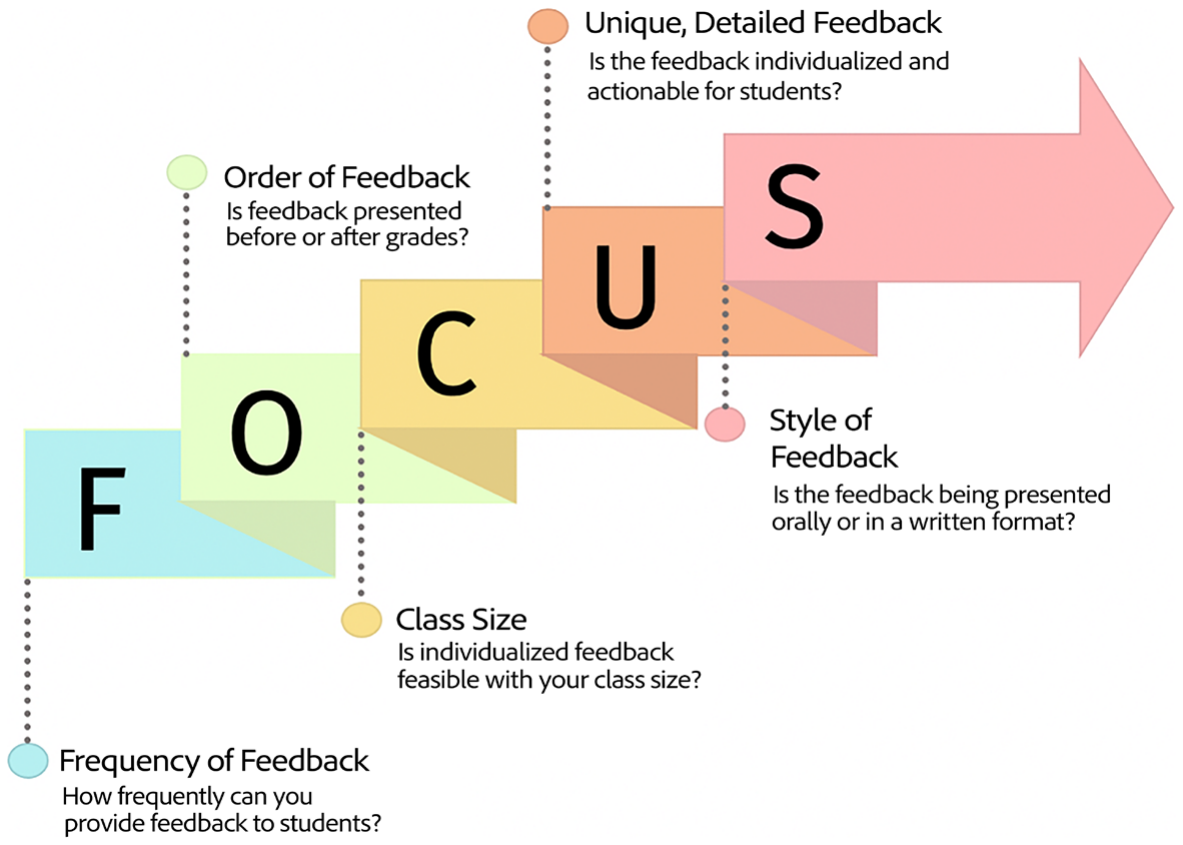
Schematic representation of considerations for communicating feedback to students in a feedback‐based formative assessment setting, using the acronym FOCUS. Brief descriptions are included for each consideration.

This study sought to examine the benefits and drawbacks of feedback‐based assessments in relation to quiz‐based assessments for graduate‐level anatomy education. Based on the findings, several crucial details related to the successful implementation of feedback‐based assessment emerged. Accordingly, the acronym FOCUS was created to summarize key considerations for communicating feedback to students, aimed at instructors looking to incorporate elements of feedback‐based assessment into formative evaluations (Figure 1).

### Frequency of feedback

The first consideration, *Frequency of Feedback*, encourages instructors to consider how often they can provide students with feedback, given the course design and potential time and resource limitations. Students in this study preferred receiving feedback at regular intervals, in this case weekly, such that its reception was a consistent element of their learning process. This is supported in the literature, with more frequent feedback being considered optimal for student learning.^69^ Despite this, providing frequent feedback to students can be time‐intensive and therefore unfavorable for instructors, which students in this study also indicated an awareness of. However, it is relevant to note that the optimal frequency of feedback can vary, based on the learning outcomes of a given course.^69^ For example, students in this study were expected to develop mastery of a large volume of content in a short period of time, hence they stood to benefit from receiving feedback on their progress frequently. Regardless of approach, instructors should clearly articulate their plan for feedback and be transparent regarding limits to the frequency with which they are able to provide feedback.

### Order of feedback

The second consideration, *Order of Feedback*, is for assessments which include components of both feedback and grading, as was the case in the feedback‐based blocks in the present study. As the provision of numeric grades is often an institutional requirement of instructors, it can be difficult to separate grades from learning and help students to make that same mental transition. Previous research has established that written feedback provided in the absence of grades improves student performance more than numeric grades or a combination of the two; however, in contexts where grades must be provided to registrars and their complete elimination is not an option, the order in which the feedback is presented relative to the grade may aid students' receptiveness to it.^70^ Indeed, students in this study reported being more receptive to their grades after reading their feedback first, and they reported that when they instead viewed their grades first, their feelings toward the feedback were more negative and they were less likely to be motivated to implement it. This finding revealed an avenue for future investigation aimed at determining whether this phenomenon exists in other contexts.

### Class size

Providing formative feedback to students can be time‐ and labor‐intensive for instructors, which is largely a factor of class size. Instructors in this study highlighted that, with a small class size of 15 students, they felt that providing feedback was more feasible than in larger classes. The students echoed an understanding of this limitation in their interviews based on their previous experiences as undergraduate students. When deciding whether to implement feedback‐based formative assessments, class size can certainly be a barrier to providing individualized feedback to students. To address this, instructors should consider whether there are other resources available to them that may assist in feedback provision. For example, instructors in courses with a laboratory or tutorial component may have teaching assistants who can be tasked with providing some or all of the feedback, thus, reducing the burden on the instructor.^71^ In courses without teaching assistants, elements of an institution's LMS may be able to be used to auto‐generate feedback for students. Hedtrich and Graulich (2018) developed software add‐ons which were compatible with various LMS applications and created formative feedback that could be automatically sent to students, highlighting areas of strength and offering suggestions for improvement based on the data available in their LMS.^72^ This example allows instructors to address large class sizes while still providing somewhat individualized support. These and other such time‐saving strategies will undoubtedly only continue to improve with the more widespread incorporation of generative artificial intelligence in recent years.^73^

### Unique feedback

Effective feedback has been described as that which is specific or individualized, as well as actionable for students, such that they can direct their own learning.^74^, ^75^ Students in the present study emphasized their preference for receiving feedback that was unique to them, highlighting their individual areas for growth. They also stated feeling less receptive to generalized feedback, as it was perceived to be less relevant to their learning, leading to students being dismissive of this type of feedback. This is supported by existing literature which suggests that indistinct praise or generalized feedback was perceived as less beneficial to students' learning than personalized feedback since the latter allowed them to determine specific areas in which to focus their studying.^76^ This effect existed when the feedback was elaborative, meaning that it provided clear and detailed explanations to students, rather than only indicating the correctness of their work.^77^ Therefore, when providing feedback, it is crucial for instructors to ensure that it is actionable, specific, and relevant to encourage students to implement it into their future work.^77^

### Style of feedback

Agreement on the optimal style of feedback, whether written or oral, can be considered a gray area in the literature. Studies exist to support the efficacy of both styles, independently; however, when they are compared, there appears to be no significant difference between the two.^78^ Some studies have found that students report a preference for oral feedback over written feedback because of the enhanced social element of speaking with the instructor compared to receiving feedback through a LMS.^74^ In contrast, some research suggests that written feedback may be more beneficial, as students who receive oral feedback may forget what they had been told and have no way of revisiting it.^78^ Students in the present study reported experiencing a similar phenomenon and, thus, preferred written feedback that they could refer back to throughout the course. In addition to this “forgetting effect,” multiple other factors can influence the effect that style of feedback has on students. For example, studies assessing part‐time versus full‐time learners determined that part‐time students benefited more from receiving feedback than full‐time learners did, regardless of style, indicating that the nature of the course and the specific student population may have some part in dictating what is an appropriate feedback delivery method.^78^ Oral feedback provided in group settings may also be influenced by a student's perception of whether the feedback is relevant to them, specifically, which may lead students to overlook the feedback if they interpret it as not being directly relevant to them.^70^ These elements should be taken into consideration, along with feasibility and logistical concerns, when determining an appropriate style of feedback.

## LIMITATIONS

One limitation of this study is that the length of each block varied from four to nine weeks, which may have influenced students' levels of motivation and academic‐related anxiety. Notably, this variation impacted the amount of time spent with each instructor, which may have had implications for the effectiveness of their teaching presence and the student's comfort levels with different instructors. For example, students typically reported increased academic‐related stress when referring to units of shorter length, which may in part be due to few interactions with the instructor. Students did not, however, indicate feeling any differences in perceived knowledge retention between blocks, which was in line with established literature on course length and student achievement and retention.^79^, ^80^ Another limitation came from the cohort studied, both in its small size and demographic distribution. The nine students interviewed represented 60% of the class (total of 15 students), and while power calculations are not appropriate for this exploratory qualitative study, this small *n*‐value may have limited the generalizability of these results to other non‐graduate populations, namely large‐sized post‐secondary classes. Additionally, the student demographic distribution was relatively homogenous: the majority identified as women, and most were around the average age of 23 years. While there is conflicting literature regarding the influence of gender on academic stress, potential gender differences related to academic stress cannot be assessed with this limited demographic distribution.^81^ Similarly, gender differences related to students' motivation could not be evaluated in this context, though other studies have demonstrated that participants who identify as women characteristically experience enhanced intrinsic motivation in education, compared to the heightened extrinsic motivation among those who identify as men.^82^ Lastly, students in this study had similar academic backgrounds (science‐related); thus, some level of content familiarity may have influenced the reported levels of academic‐related stress, as Schönwetter and colleagues (2002) demonstrated that content‐unfamiliar students relied more heavily on instructor organization, while content‐familiar students were more aware of instructor expressiveness.^83^

## CONCLUSION AND FUTURE DIRECTIONS

Alternative assessment practices, such as feedback‐based assessment, provide a means for instructors to enhance student engagement and motivation while also mitigating students' stress related to traditional grading. While many of these practices have been implemented across disciplines and educational levels, most are used in summative undergraduate evaluation settings. The results of this study offer insights into both student and instructor perceptions on implementation of feedback‐based assessments in a formative setting as part of a graduate anatomy education program. Student interviews emphasized the critical importance of clear communication of the instructor's goals and expectations, as well as how a lack of communication could result in a disconnect between perceived effort and outcomes, leading to enhanced stress and decreased motivation. Instructor interviews demonstrated their intentions to promote individualized assessment in authentic learning environments, while also developing NTDIS through their evaluations. Taken together, these perceptions highlight the importance of intentional, constructively aligned course design and clear communication between instructors and students, especially as it relates to the effective implementation of feedback‐based assessment practices. Additional focus should be paid to the influence of student demographics, including but not limited to age, gender, race, and previous academic background, on student–instructor interactions, which could subsequently impact students' stress and motivation.

## AUTHOR CONTRIBUTIONS

**Emily L. Dietrich:** Conceptualization; data curation; formal analysis; investigation; methodology; visualization; writing – original draft; writing – review and editing. **Sean C. McWatt:** Conceptualization; funding acquisition; investigation; methodology; project administration; resources; software; supervision; writing – review and editing.

## Supporting information


**Figure S1.**.

**Data S1.**.
