# Supplementary figures and images for: Exploring perceptions of alternative assessment and grading in graduate anatomy education
Source: Anat Sci Educ. 2024 Dec 31;18(2):172–91. doi: 10.1002/ase.2550 (PMC11797536; doi:10.1002/ase.2550)

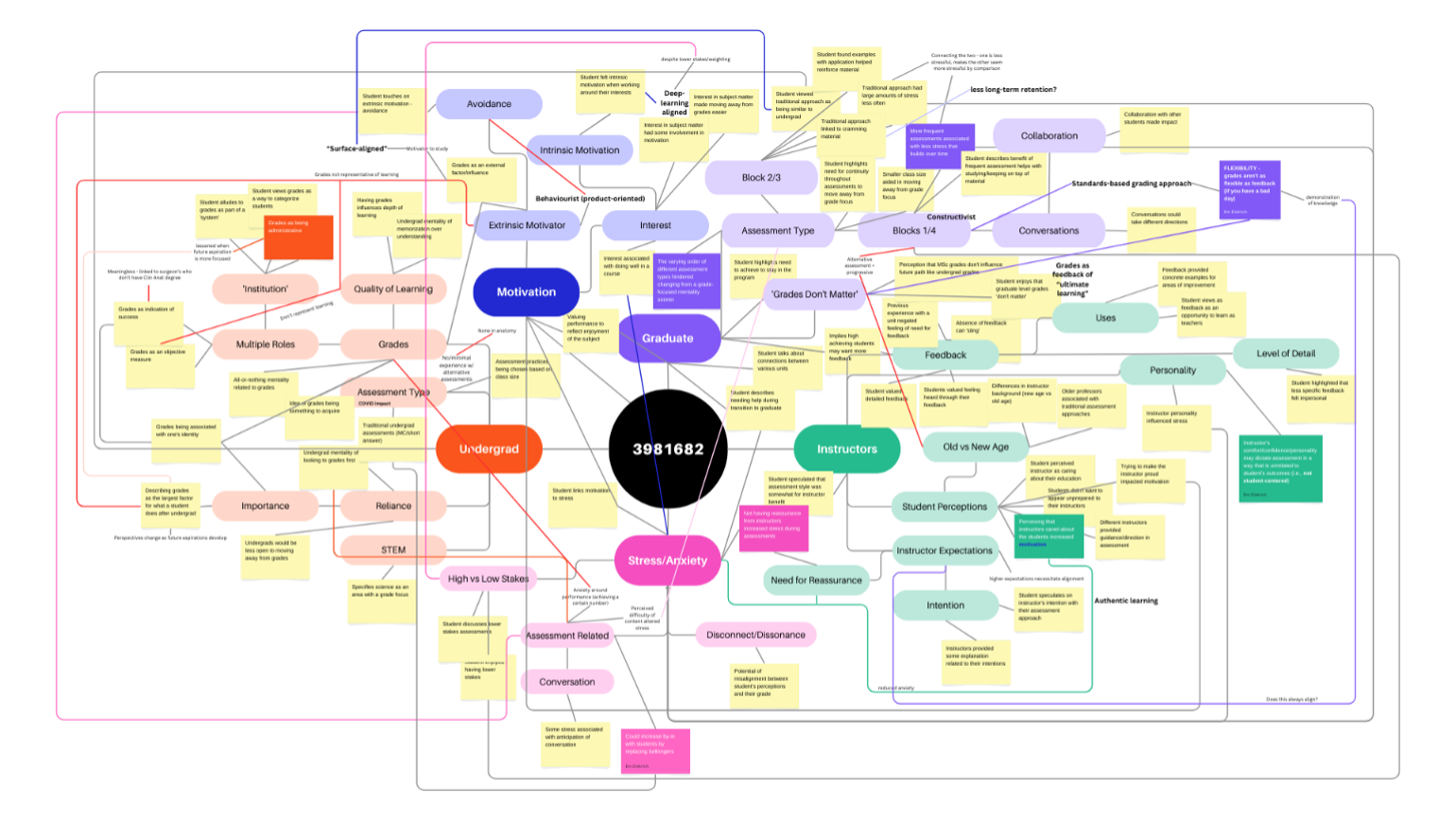

Supplement: Supplementary file 1 — Figure S1.. [file ASE-18-172-s002.tiff]
