# Supplementary material for: Exploring perceptions of alternative assessment and grading in graduate anatomy education
Source: Anat Sci Educ. 2024 Dec 31;18(2):172–91. doi: 10.1002/ase.2550 (PMC11797536; doi:10.1002/ase.2550)
Supplement: Supplementary file 2 — Data S1.. [file ASE-18-172-s001.docx]

**SUPPLEMENTAL DIGITAL APPENDICES**

***Supplemental Digital Appendix 1:*** *11 Competencies and Brief Descriptors (Block 4)*

1. Regional Anatomy.
   1. Basic anatomy, identification, function, and terminology. Connecting Block 4 content with previous content from the course.
2. Referred Pain
   1. Explanation of referred pain and/or their pathways.
3. Fascial Planes
   1. Leveraging fascial planes to approach a dissection or regionalize a structure
4. Spatial Ability
   1. Anatomical relationships, predicting the location of undissected structures, navigating different orientations and perspectives.
5. Embryology
   1. Explain a finding or observation using an embryological basis.
6. Research
   1. Bring primary literature into discussions in order to inform an answer or explain a finding.
7. Dissection Approach
   1. Conceive and justify a dissection approach that deviates from the laboratory manual, then execute it.
8. Instrumental Proficiency
   1. Selection and handling of tools, dissecting with intention, suturing/knot-tying skills.
9. Flipped Learning
   1. Role reversal: create a *viva* question for the instructor.
10. Anatomical Demonstration
    1. Teach/demonstrate a core concept to a pretend/lay audience (e.g., secondary school students).
11. Comradery and Collaboration
    1. Typically an observation from afar. Interaction with group members and investment in their learning.

***Supplemental Digital Appendix 2****: Semi-Structured Interview Questions – Students*

1. Thinking of your experience in education in general, what are your thoughts on how grades and graded assessments influence your learning process and learning outcomes in your courses?
2. How do you feel about the purpose or role of grades in learning?
3. How do graded assessments influence your own experience as a learner?
4. What role do grades and graded assessments have in your motivation to learn?
5. Do you think that grades and having graded assessments impacts the quality of your learning outcomes? How?
6. What about test anxiety – do you typically experience assessment-related stress or anxiety? Please describe whatever you are comfortable sharing.
7. What about grades, specifically – can you describe how grades and graded assessments impact your anxiety? Please be as specific as possible with what you are comfortable sharing.
8. Throughout your education, have you experienced any courses or assessment practices that didn’t rely or minimally relied on grades? Please describe in as much detail as possible.
   1. How did those courses or assessments impact your learning experience?
   2. Did these types of assessments influence your motivation to learn? How?
   3. What about test anxiety – did the assessments with decreased or no grading impact your feelings of anxiety in any way?
   4. In terms of overall learning outcomes, how do you think these types of assessments influenced the quality of your learning?
9. Thinking of the 9560 Gross Anatomy course specifically, how do you feel about the role of grades and the assessments used throughout the duration of course?
10. Did you notice any differences in the assessment practices used in 9560 compared to your previous course experiences? What differences did you notice? What similarities did you notice? Please be as specific as possible.
11. How did the assessment practices used in this course influence your learning experience compared to assessment practices used in previous courses?
12. What about motivation – did the assessments in this course have a different impact on your motivation to learn than those in previous courses?
13. Did you experience any stress or anxiety, specifically related to the assessments and grading practices in this course? Please describe in as much detail as you feel comfortable sharing.
14. Looking back on all of the grading and assessment approaches used in 9560, how do you think the quality of your anatomy learning was impacted relative to previous courses?
15. Did you notice any differences in the assessment practices used between the different instructors or units in the 9560 Gross Anatomy course?
16. What differences did you notice? What similarities did you notice? Please be as specific as possible.
17. With relation to the assessment practices used by each instructor in this course, did you notice any differences in grading or the influence of grades? If so, please describe how that influenced your learning experience and learning outcomes.
18. Do you have a preference for one type of assessment or grading practice used in this course over another? If so, why?
19. Do you feel that your motivation to learn the content was impacted by one type of assessment or grading practice over another? If so, how?
20. Do you feel that your levels of assessment-related stress were impacted differently between assessment and grading practices in this course? Please describe in as much detail as you are comfortable sharing.
21. Regarding your overall learning in the course, do you feel that the quality of your learning was impacted by one type of assessment or grading practice over another? If so, how?
22. How did the instructor, themselves (i.e., who they are, their personality, etc.), impact your experience with the different assessments? Did that make a difference? Could *any* instructor have delivered the assessment(s) to the same effect?
23. How did the instructor, themselves (i.e., who they are, their personality, etc.), impact the influence that the assessments and grading practices had on your motivation to learn and the quality of your learning outcomes?
24. What about anxiety – did the instructor, themselves (i.e., who they were, their personality, etc.), influence your assessment-related anxiety?
25. Are there any other comments or questions related to grading, assessment, learning motivation, or assessment-based anxiety that you wish to share with the research team?

***Supplemental Digital Appendix 3****: Semi-Structured Interview Questions – Instructors*

1. Please describe how you approached assessment and grading in this gross anatomy course.
   1. How might this differ from your approach to assessment and grading in other populations you teach?
   2. Have you adapted your approach to assessment and grading over time? How?
   3. Do you intend to make any changes to your assessment and grading approaches in the future? Please describe?
2. Thinking about your block of the course specifically, please describe the pedagogical philosophy or philosophies that informed your choice of grading and assessment strategies for the block assessments specifically, in addition to the final oral *viva*.
3. Were these philosophies informed from your own educational experience?
4. Have you explored any literature that has informed your approach to grading and assessment?
5. Please describe the intention(s) behind your chosen grading and assessment strategies and what goals you had, if any, for students’ learning through the implementation of these strategies.
   1. Did you have a ‘hidden curriculum’ associated with your assessment and grading practices?
6. How effective do you feel that your grading and assessment strategies were throughout your block (i.e., how do you think the assessments aligned with your goals or intentions for the course and how did the assessments impact students’ learning outcomes, etc.)?
   1. How do you think your assessment practices influenced student motivation?
   2. How do you think your practices influenced assessment-related anxiety experienced by your students?
7. Do you have any other comments related to your grading and assessment strategies that you would like to share with the study team?

***Supplemental Digital Appendix 4:*** *Example of Participant Profile*

**Supporting Information Material Figure 1.** Thematic map generated as part of creation of a participant profile. The yellow boxes contain codes from the associated student transcript [3981682], which were then grouped into themes and associated subthemes as part of the data familiarization process, as outlined by Braun and Clarke (2006).
